# Elevated Serum Inflammatory Markers in Subacute Stroke Are Associated With Clinical Outcome but Not Modified by Aerobic Fitness Training: Results of the Randomized Controlled *PHYS-STROKE* Trial

**DOI:** 10.3389/fneur.2021.713018

**Published:** 2021-08-26

**Authors:** Bernadette Kirzinger, Andrea Stroux, Torsten Rackoll, Matthias Endres, Agnes Flöel, Martin Ebinger, Alexander Heinrich Nave

**Affiliations:** ^1^Center for Stroke Research Berlin, Charité – Universitätsmedizin Berlin, Berlin, Germany; ^2^Institute of Biometry and Clinical Epidemiology, Charité – Universitätsmedizin Berlin, Berlin, Germany; ^3^Berlin Institute of Health QUEST Center for Transforming Biomedical Research Berlin, Charité – Universitätsmedizin Berlin, Berlin, Germany; ^4^NeuroCure Clinical Research Center, Charité – Universitätsmedizin Berlin, Berlin, Germany; ^5^Klinik Und Hochschulambulanz für Neurologie, Charité – Universitätsmedizin Berlin, Berlin, Germany; ^6^German Center for Cardiovascular Research, Partner Site Berlin, Berlin, Germany; ^7^German Center for Neurodegenerative Diseases, Partner Site Berlin, Berlin, Germany; ^8^Berlin Institute of Health at Charité – Universitätsmedizin Berlin, Berlin, Germany; ^9^Department of Neurology, University Medicine Greifswald, Greifswald, Germany; ^10^German Center for Neurodegenerative Diseases, Partner Site Rostock/Greifswald, Greifswald, Germany; ^11^Medical Park Berlin Humboldtmühle, Berlin, Germany

**Keywords:** stroke, inflammation, outcome, IL-6, crp, TNF-alpha, fibrinogen, biomarkers

## Abstract

**Background:** Inflammatory markers, such as C-reactive Protein (CRP), Interleukin-6 (IL-6), tumor necrosis factor (TNF)-alpha and fibrinogen, are upregulated following acute stroke. Studies have shown associations of these biomarkers with increased mortality, recurrent vascular risk, and poor functional outcome. It is suggested that physical fitness training may play a role in decreasing long-term inflammatory activity and supports tissue recovery.

**Aim:** We investigated the dynamics of selected inflammatory markers in the subacute phase following stroke and determined if fluctuations are associated with functional recovery up to 6 months. Further, we examined whether exposure to aerobic physical fitness training in the subacute phase influenced serum inflammatory markers over time.

**Methods:** This is an exploratory analysis of patients enrolled in the multicenter randomized-controlled *PHYS-STROKE* trial. Patients within 45 days of stroke onset were randomized to receive either four weeks of aerobic physical fitness training or relaxation sessions. Generalized estimating equation models were used to investigate the dynamics of inflammatory markers and the associations of exposure to fitness training with serum inflammatory markers over time. Multiple logistic regression models were used to explore associations between inflammatory marker levels at baseline and three months after stroke and outcome at 3- or 6-months.

**Results:** Irrespective of the intervention group, high sensitive CRP (hs-CRP), IL-6, and fibrinogen (but not TNF-alpha) were significantly lower at follow-up visits when compared to baseline (*p* all ≤ 0.01). In our cohort, exposure to aerobic physical fitness training did not influence levels of inflammatory markers over time. In multivariate logistic regression analyses, increased baseline IL-6 and fibrinogen levels were inversely associated with worse outcome at 3 and 6 months. Increased levels of hs-CRP at 3 months after stroke were associated with impaired outcome at 6 months. We found no independent associations of TNF-alpha levels with investigated outcome parameters.

**Conclusion:** Serum markers of inflammation were elevated after stroke and decreased within 6 months. In our cohort, exposure to aerobic physical fitness training did not modify the dynamics of inflammatory markers over time. Elevated IL-6 and fibrinogen levels in early subacute stroke were associated with worse outcome up to 6-months after stroke.

**Clinical Trial Registration:**ClinicalTrials.gov, NCT01953549.

## Introduction

Over the past decades, progress in stroke treatment has led to lower mortality rates but at the same time, increased numbers of stroke survivors implicate an increasing need for post-stroke rehabilitation. Stroke is the third leading cause of Disability Adjusted Life Years; 40% of stroke survivors are disabled between 1 month and 5 years after the event (1). It is therefore crucial to classify ways that improve outcome after stroke.

Biomarkers have become a relevant topic in stroke research and multiple interesting markers have been identified (1). Amongst them, inflammatory biomarkers have been found to affect stroke etiology and outcome (2). In heart disease, an association of upregulated inflammation with a higher risk of recurrent events and impaired outcome is widely acknowledged (3, 4). Similar associations have been found for stroke patients (4). In the post-stroke brain, dying neuronal cells release damage signals and danger-associated molecular patterns are exposed, subsequently activating microglia and peripheral leucocytes, which both release inflammatory cytokines as a response (5). However, little is known about the long-term time course of inflammatory parameters after stroke (6–10). Most studies investigate inflammatory activity in the acute phase after stroke, only a few provide long-term measurements (9). Some studies suggest that elevated inflammatory markers are associated with the risk of recurrent events and poorer outcome after stroke and lead to poorer outcome (11, 12). Approaches of measuring inflammatory biomarkers to predict stroke outcome in the clinical setting have been discussed (1, 13–15). Their final role in stroke pathogenesis and functional recovery, however, remains uncertain (12, 16). While it has deleterious effects on the post-stroke brain, inflammation is crucial for post-stroke tissue recovery and neovascularization (17). Up to date, no effective ways to downregulate inflammatory activity have been established in the clinical setting (18, 19). Physical fitness training may downregulate inflammatory processes in the long term (20).

We investigated the dynamics of high-sensitive C-reactive protein (hs-CRP), interleukin-6 (IL-6), tumor necrosis factor-alpha (TNF-alpha) and fibrinogen in the subacute phase following stroke. With our intervention group receiving a 4-week fitness training program, we explored whether aerobic physical fitness training in the subacute phase is associated with the course of inflammatory biomarkers. Furthermore, we determined if fluctuations in inflammatory markers are associated with functional outcome after stroke.

## Methods

### Study Participants and Trial Design

This exploratory analysis is part of an observational biomarker sub-study nested within the multicenter, randomized controlled, endpoint blinded *PHYS-STROKE* trial. The details of the *PHYS-STROKE* study design and the main results have been published previously (21, 22). In total, 200 patients in the subacute phase of ischemic or hemorrhagic stroke (5–45 days after the index event) were recruited at 7 rehabilitation centers in and around Berlin, Germany, between 2013 and 2017. Patients were randomized 1:1 to receive either 4 weeks of treadmill-based, aerobic physical fitness training or a 4-week relaxation program in addition to standard rehabilitation therapy. For inclusion into the trial, patients had to be at least 18 years of age, present with a baseline Barthel Index (BI) of 65 or less and be capable of participating in an aerobic fitness program. In the clinical setting, the BI is a widely used tool to measure impairments in activities of daily living (23). Patients were excluded if they needed assistance in walking before stroke, were unable to sit unsupported for at least 30 s or had severe psychiatric or cardiac comorbidities. A full list of inclusion and exclusion criteria to the *PHYS-STROKE* study can be found in Supplementary Table 1. If patients met the eligibility criteria, written informed consent was obtained. During the intervention, all additional therapy sessions were recorded and therapists and patients were instructed to note the time of additional therapy they received outside of their participation in the *PHYS-STROKE* study (22). No standard treatment policy was set for the post-intervention period (22). Aerobic fitness training was performed using treadmill-based training to achieve a target heart rate using the formula “180 min years of age”. A period of 4 weeks was chosen to ensure that intervention session took place during the in-patient stay at the rehabilitation centers (21). A subsample of patients were additionally enrolled in the accompanying observational biomarker study *BAPTISe* (“Biomarkers And Perfusion – Training-Induced Changes After Stroke”) and received additional blood biomarker measurements and MRI scans before and after the intervention (24). *PHYS-STROKE* participants with at least baseline inflammatory marker measurements were included in this analysis. Ethical approval was obtained from the institutional review board of Charité–Universitätsmedizin Berlin (*PHYS-STROKE*: EA1/138/13; *BAPTISe*: NCT01954797).

### Follow-Up Visits and Outcomes

Clinical follow-up visits took place at study enrollment (baseline), after the 4-week trial intervention period (v1), as well as 3- (v2) and 6-months (v3) after stroke. Screening of patients was performed by the trial physician of the respective site. Study outcomes were assessed and documented by trained study assessors at each visit. Study assessors and the trial statistician were blinded to the intervention. Detailed descriptions of outcome parameters were reported previously (21, 22). For this project, functional outcome was analyzed using the modified Rankin Scale (mRS), BI and maximal walking speed (MWS) at 3 and 6 months after stroke. The mRS assesses the degree of handicap in the clinical setting (25).

### Measurement of Inflammatory Markers

Fasting blood samples were retrieved at the recruiting rehabilitation center at each follow-up visit. Laboratory analyses of serum inflammatory markers were performed within 6 h at the “Labor Berlin” in Berlin, Germany. Blood levels of hs-CRP, IL-6 and TNF-alpha were determined using solid-phase, chemiluminescent immunometric assays (IMMULITE® 1,000, Siemens Healthcare Diagnostics). Measurement of fibrinogen levels was performed based on the Clauss method in citrated blood plasma by using the HemosIL® Q.F.A. Thrombin (Bovine) kit, Instrumentation Laboratories. Standard laboratory reference values were used to define elevated serum levels for each inflammatory marker: <3.0 mg/l for hs-CRP, <3.8 ng/l for IL-6, <8.1 pg/ml TNF-alpha, and 2–4 g/l for fibrinogen.

### Statistical Analysis

Primary research questions of this project were the dynamics of inflammatory markers over a time course of 6 months and differences in inflammatory biomarkers between the two treatment groups. Secondary aims were the associations of inflammatory markers at baseline with functional outcome parameters at 3- and 6-months after stroke.

Using Generalized estimating equation (GEE)-models, we investigated the dynamics of inflammatory markers, as well as associations of levels of inflammatory markers over time with respective patient characteristics and cerebrovascular risk factors. GEE-models allow estimating associations for repeated measurements (26). We used an exchangeable correlation matrix and calculated models for each biomarker individually. For GEE-models, we used levels of inflammatory markers over time (from baseline to v3) as dependent variables. As independent variables we chose a list of clinically relevant baseline parameters and cerebrovascular risk factors: center, visits/time, age, sex, intervention group, TOAST-classification, baseline NIHSS, smoking, atrial fibrillation, diabetes mellitus, arterial hypertension, cerebro- and cardiovascular disease. Cerebrovascular disease was defined as previous stroke and/or transient ischemic attack (TIA). Cardiovascular disease was defined as history of chronic heart disease (CHD), peripheral artery disease (PAD) and/or myocardial infarction (MI).

We used multivariate logistic regression models to analyze associations between levels of inflammatory biomarkers at baseline and 3 months after stroke and functional outcome parameters up to 6 months. Functional outcome parameters (mRS, BS and MWS) were dichotomized by the median and used as dependent variables in the multivariate models. As independent variables, we used absolute levels of single inflammatory markers (as continuous variables). Furthermore, we added sex, National Institutes of Health Stroke Scale (NIHSS) scores at baseline and age, as well as arterial hypertension (*p* ≤ 0.1 in univariate analyses) to our models. Multivariate models were calculated using backward selection.

For all statistical analyses we used SPSS Version 25 and 27. Non-normally distributed data were log-transformed. *P*-values ≤ 0.05 are considered significant. Due to the exploratory character of the study, no Bonferroni correction has been performed.

## Results

### Baseline Characteristics of the PHYS-STROKE Cohort

Between September 2013 and April 2017, 200 participants were enrolled in the *PHYS-STROKE* study of which 105 (53%) were randomized in the fitness and 95 (48%) in the relaxation group. The mean age of the study cohort was 69 years (SD ± 12) and 119 (60%) of our participants were male. One hundred eighty-one (90.5%) participants suffered from ischemic stroke, 19 (9.5%) from hemorrhagic stroke. At study enrollment, our cohort presented with a median NIHSS of 8 (IQR: 5–12), a median mRS of 4 (IQR: 4.00–4.00), a median BI of 50 (IQR: 35–60) and a median MWS of 0.30 m/s (IQR: 0.13–0.66 m/s). No significant differences between the two groups regarding the baseline characteristics were determined. Table 1 shows a detailed list of baseline characteristics of the study cohort.

**Table 1:** Baseline characteristics of the *PHYS-STROKE* cohort.

	**All (*n* = 200)**	**Fitness (*n* = 105)**	**Relaxation (*n* = 95)**	***p*-value**
Age (years, mean ± SD)	69 ± 12	69 ± 12	70 ±11	0.53
Male (*n*, %)	119 (59.5)	60 (57.1)	59 (62.1)	0.56
Ischemic stroke (*n*, %)	181 (90.5)	91 (86.7)	90 (94.7)	0.06
TOAST (*n*, %)				0.86
Large-artery atherosclerosis	36 (18.0)	17 (18.7)	19 (21.3)	
Cardioembolic	36 (18.0)	18 (19.8)	18 (20.2)	
Small-vessel occlusion	30 (15.0)	16 (17.6)	14 (15.7)	
Other determined etiology	7 (3.5)	3 (3.3)	4 (4.5)	
Undetermined etiology	62 (31.0)	34 (37.4)	28 (31.5)	
Two or more causes identified	9 (4.5)	3 (3.3)	6 (6.7)	
NIHSS [median (IQR)]; missing 1^*^	8 (7)	9 (7)	7 (6)	0.29
mRS [median (IQR)]	4 (4)	4 (4)	4 (3,4)	0.15
BI [median (IQR)]	50 (35–60)	50 (35–60)	55 (35–65)	0.19
MWS [m/s, median (IQR)]; missing 4	0.30 (0.13–0.66)	0.22 (0.13–0.56)	0.38 (0.14–0.71)	0.12
Stroke to intervention (days, mean ± SD); missing 4^+^	32.36 ± 40.44	28.06 ± 12.96	37.10 ± 56.88	0.77
Smoking until stroke (*n*, %); missing 1	63 (31.5)	32 (30.5)	31 (32.6)	0.94
Diabetes Mellitus (*n*, %)	63 (31.5)	32 (30.5)	31 (32.6)	0.76
Arterial hypertension (*n*, %)	166 (83.0)	86 (81.5)	80 (84.2)	0.71
Atrial fibrillation (*n*, %)	46 (23.0)	23 (21.9)	23 (24.2)	0.74
Hypercholesterolemia	80 (40.0)	43 (41.0)	37 (38.9)	0.89
Cardiovascular disease (CHD, MI, PAD; *n*, %)	34 (17.0)	13 (12.4)	21 (22.1)	0.09
CHD	29 (14.5)	11 (10.5)	18 (18.9)	
MI (> 120 days)	2 (1.0)	1 (1.0)	1 (1.1)	
PAD	10 (5.0)	4 (3.8)	6 (6.3)	
Cerebrovascular disease (stroke, TIA; *n*, %)	54 (27.0)	27 (25.7)	27 (28.4)	0.75
Stroke	38 (19.0)	20 (19.0)	18 (18.9)	
TIA	22 (11.0)	11 (10.5)	11 (11.6)	
hs-CRP at baseline (mg/l, mean ± SD); missing 5	12.04 ± 18.72	12.05 ± 18.69	12.03 ± 18.85	0.57
IL-6 at baseline (pg/ml, mean ± SD); missing 3	6.34 ± 11.72	5.70 ± 6.80	7.04 ± 15.43	0.59
TNF-alpha at baseline (pg/ml, mean ± SD); missing 7	9.34 ± 3.85	8.96 ± 3.68	9.77 ± 4.00	0.10
Fibrinogen (g/l, mean ± SD); missing 7	4.09 ± 1.15	4.12 ± 1.29	4.06 ± 0.98	0.85

*TOAST, Trial of Org 10172 in Acute Stroke Treatment; NIHSS, National Institutes of Health Stroke Scale; mRS, modified Rankin Scale; BI, Barthel Index; MWS, Mean Walking Speed; CHD, Coronary heart disease; MI, Myocardial infarction; PAD, Peripheral artery disease; TIA, Transient ischemic attack; hs-CRP, high-sensitive C-reactive protein; IL-6, Interleukin 6; TNF-alpha, Tumor Necrosis Factor alpha*.

* *Hospital chart missing*.

+ *Excluded at screening*.

### Baseline Inflammatory Markers and Dynamics of Inflammatory Markers

**Figure 1:**
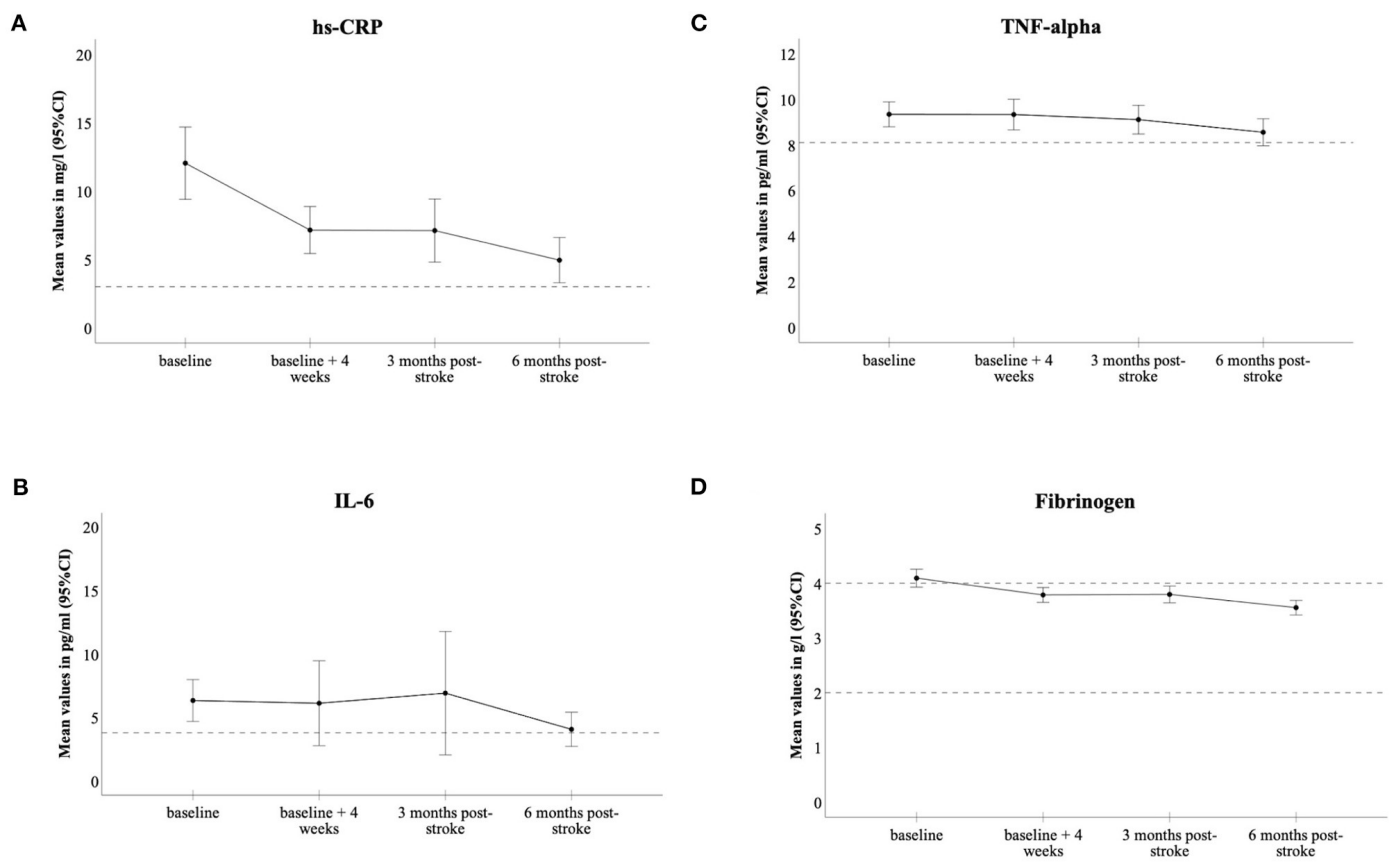
Dynamics of inflammatory biomarkers in the PHYS-Stroke study cohort; hs-CRP **(A)**, IL-6 **(B)**, TNF-alpha **(C)**, fibrinogen **(D)**. Levels of markers are expressed as means, 95% CIs as error bars; horizontal dashed lines depict cut-off for normal ranges.

The median time from stroke onset to the start of intervention was 28 days (IQR: 17–40). At baseline, the mean levels of all investigated inflammatory markers were above the clinical cut-offs. The mean serum concentrations of inflammatory markers showed a falling tendency over the 6-month observation period. The mean values of hs-CRP, IL-6, TNF-alpha, and fibrinogen over a time course of 6 months are depicted in Figure 1. In GEE-models, hs-CRP, IL-6, and fibrinogen were significantly lower at all follow-up visits when compared to baseline visits (*p* < 0.01 for all three inflammatory markers and all time points from v1 to v3 as compared to baseline). TNF-alpha was elevated post-stroke and declined over the study period; however, no statistically significant decline was observed (v1: Coef. 0.01; v2: Coef. −0.001; v3: Coef. −0.02; *p*-values between 0.09 and 0.9).

### Associations of Risk Factors With Level of Inflammatory Biomarkers Over Time

GEE models demonstrated that elevating hs-CRP (Coef. 0.01, *p* = 0.03), IL-6 (Coef. 0.01, *p* < 0.01) and TNF-alpha (Coef. 0.004, *p* < 0.01) levels over time correlated with increasing age. Additionally, levels of inflammatory markers over time were associated with prevalent cardiovascular risk factors at baseline: participants with pre-existing arterial hypertension had significantly increased levels of hs-CRP (Coef. 0.23, *p* = 0.04) and smokers had significantly elevated hs-CRP (Coef. 0.18, *p* = 0.04) and IL-6 (Coef. 0.09, *p* = 0.04) levels over the course of 6 months after stroke. Participants presenting with a history of cardiovascular, but not cerebrovascular, disease had significantly increased TNF-alpha levels over time (Coef. 0.07, *p* = 0.02), compared to participants without cardiovascular disease. Fibrinogen levels over time did not show any associations with investigated risk factors in GEE-models. Table 2 shows an overview of the results of the GEE-models.

**Table 2:** Factors associated with levels of inflammatory biomarkers over time; GEE-models.

	**hs-CRP**		**IL-6**		**TNF-alpha**		**Fibrinogen**	
	**Coef**.	**95% CI**	**Coef**.	**95% CI**	**Coef**.	**95% CI**	**Coef**.	**95% CI**
Centre (reference: centres with early rehabilitation)	−0.14	(−0.31, 0.03)	−0.13^*^	(−0.21, −0.04)^*^	0.04	(−0.01, 0.09)	−0.2	(−0.40, 0.17)
Time (reference: baseline)								
baseline + 4 weeks	−0.20^**^	(−0.29, −0.10)^**^	−0.10^**^	(−0.16, −0.05)	0.01	(−0.01, 0.03)	−0.33^**^	(−0.50, −0.17)^**^
3 months post–stroke	−0.26^**^	(−0.36, −0.16)^**^	−0.12^**^	(−0.17, −0.06)	−0.001	(−0.023, 0.02)	−0.31^**^	(−0.50, −0.13)^**^
6 months post-stroke	−0.47^**^	(−0.57, −0.38)^**^	−0.19^**^	(−0.25, −0.13)	−0.02	(−0.04, 0.003)	−0.59^**^	(−0.76, −0.42)^**^
Age	0.01^*^	(0.00, 0.02)^*^	0.01^**^	(0.01, 0.02)	0.004^*^	(0.002, 0.01)^**^	0.01	(0.00, 0.03)
Gender (reference: male)	0.041	(−0.11, 0.19)	−0.03	(−0.11, 0.05)	0.01	(−003, 0.05)	−0.04	(−0.28, 0.20)
Intervention group (reference: relaxation)	−0.04	(−0.19, 0.10)	0.004	(−0.07, 0.08)	−0.02	(−0.06, 0.02)	−0.03	(−0.26, 0.20)
TOAST–classification (reference: undetermined etiology)								
Large–artery atherosclerosis	0.12	(−0.12, 0.35)	0.11	(−0.001, 0.22)	0.03	(−0.04, 0.09)	0.09	(−0.23, 0.41)
Cardioembolic	−0.12	(−0.50, 0.26)	0.12	(−0.20, 0.44)	0.02	(−0.07, 0.10)	−0.20	(−0.81, 0.40)
Small–vessel occlusion	−0.001	(−0.22, 0.22)	0.10	(−0.03, 0.22)	−0.004	(−0.06, 0.05)	0.35	(−0.43, 0.74)
Other determined etiology	−0.07	(−0.56, 0.42)	0.07	(−0.11, 0.25)	0.05	(−0.04, 0.13)	0.07	(−0.60, 0.74)
Two or more causes identified	−0.19	(−0.65, 0.28)	−0.03	(−0.34, 0.28)	−0.04	(−0.14, 0.07)	0.08	(−0.57, 0.71)
NIHSS at baseline	0.01	(−0.01, 0.03)	−0.002	(−0.01, 0.01)	−0.002	(−0.01, 0.003)	0.03	(−0.002, 0.05)
Smoking until stroke (reference: no smoking)	0.18^*^	(0.01, 0.35)^*^	0.09^*^	(0.002, 0.17)^*^	0.01	(−0.03, 0.06)	0.22	(−0.03, 0.48)
Atrial fibrillation (reference: no Atrial fibrillation)	0.23	(−0.14, 0.60)	−0.03	(−0.33, 0.27)	0.02	(−0.05, 0.10)	−0.04	(−0.62, 0.55)
Diabetes Mellitus (reference: no Diabetes Mellitus)	−0.10	(−0.27, 0.08)	0.04	(−0.05, 0.12)	0.04	(−0.02, 0.08)	0.09	(−0.18, 0.35)
Arterial hypertension (reference: no Arterial hypertension)	0.23^*^	(0.01, 0.45)^*^	−0.001	(−0.09, 0.09)	0.02	(−0.05, 0.08)	0.24	(−0.03, 0.51)
Cardiovascular disease (CHD, MI, PAD; reference: no Cardiovascular disease)	0.13	(−0.06, 0.32)	0.08	(−0.05, 0.20)	0.07^*^	(0.01, 0.12)^*^	0.12	(−0.21, 0.44)
Cerebrovascular disease (Stroke, TIA; reference: no Cerebrovascular disease)	0.02	(−0.17, 0.21)	0.06	(−0.05, 0.18)	−0.02	(−0.07, 0.03)	0.03	(−0.24, 0.30)

* *p < 0.05;*

** *p < 0.001*.

*hs-CRP, high-sensitive C-reactive protein; IL-6, Interleukin 6; TNF-alpha, Tumor Necrosis Factor alpha; TOAST, Trial of Org 10172 in Acute Stroke Treatment; NIHSS, National Institutes of Health Stroke Scale; CHD, Coronary heart disease; MI, Myocardial infarction; PAD, Peripheral artery disease; TIA, Transient ischemic attack*.

### Effect of Physical Fitness Training on Inflammatory Markers

**Figure 2:**
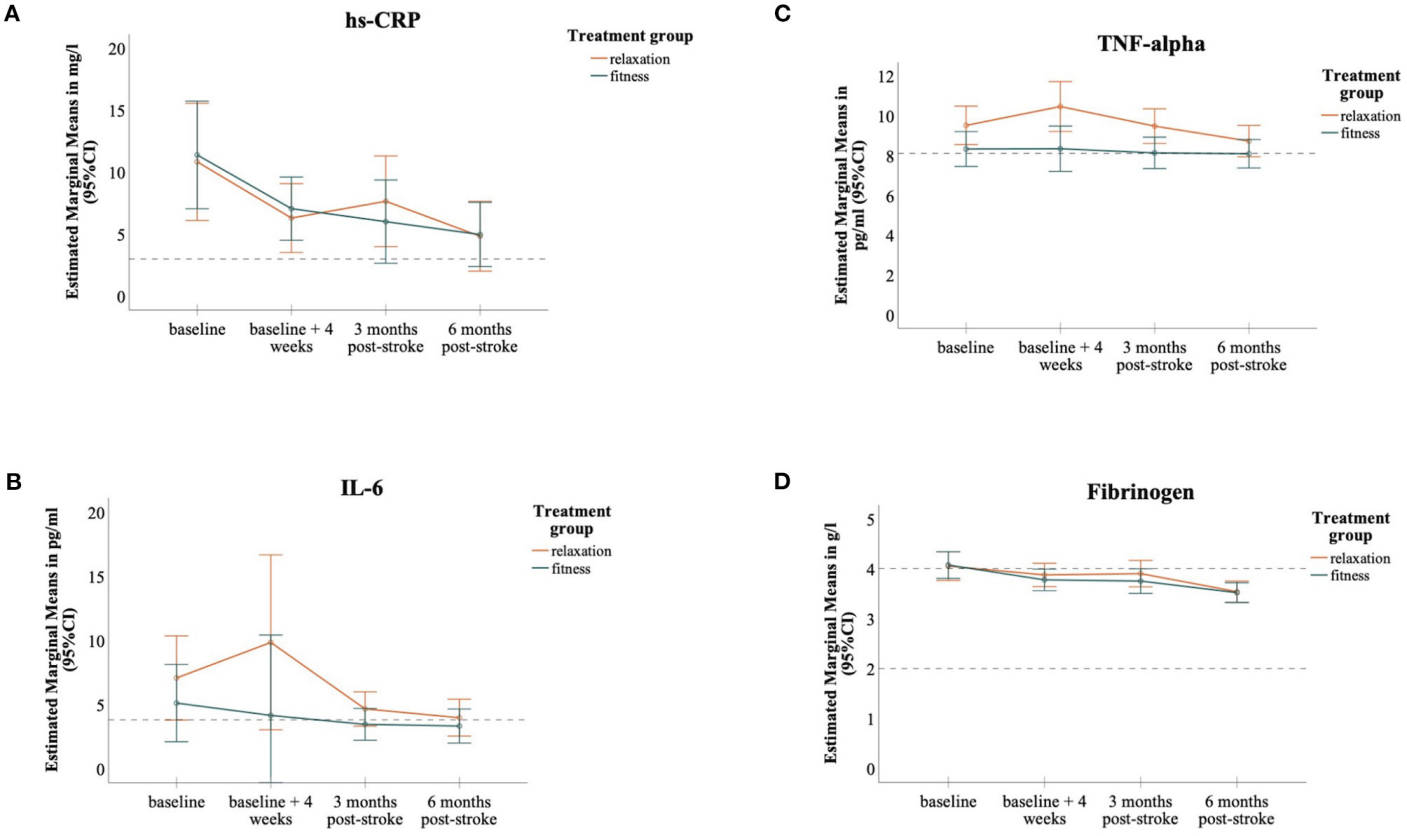
MANOVA-Models, Dynamics of inflammatory biomarkers separated into the two treatment groups; hs-CRP **(A)**, IL-6 **(B)**, TNF-alpha **(C)**, fibrinogen **(D)**; Levels of markers are expressed as Estimated Marginal Means, 95% CIs as error bars; horizontal dashed lines depict cut-off for normal ranges.

The dynamics of inflammatory biomarkers over the 6-month observation period separated by treatment groups are presented in Figure 2. In GEE-models, absolute levels of investigated inflammatory markers over a time course of 6 months did not significantly differ between the two treatment groups of *PHYS-STROKE* (hs-CRP: Coef. −0.04; IL-6: Coef. 0.004; TNF-alpha: Coef. −0.02; fibrinogen: Coef. −0.03; *p*-values between 0.29 and 0.91).

### Association of Inflammatory Markers With Outcome

In univariate models, increased baseline levels of all investigated markers were associated with worse functional outcome measurements at 3 or 6 months after stroke. Median splits of functional outcome parameters and the results of univariate analyses can be found in the Supplementary Material (Supplementary Tables 3, 4). Table 3 shows an overview of all results from multivariate logistic regression models. In multivariate logistic regression analyses, increased levels of IL-6 and fibrinogen at baseline were associated with a lower MWS (MWS ≤ 0.57 m/s) 6-months after stroke, independently of age, sex, baseline NIHSS and arterial hypertension [OR 0.34, 95% CI (0.13, 0.88); OR 0.70, 95% CI (0.51, 0.96), respectively]. Elevated baseline IL-6 and fibrinogen were also independently associated with a worse mRS (mRS ≥ 3) at 6 months after stroke [OR 3.02, 95% CI (1.01, 9.08); OR 1.59, 95% CI (1.08, 2.35), respectively]. In addition, elevated baseline levels of fibrinogen were associated with a lower BI (BI ≤ 90) 6 months post-stroke [OR 0.66, 95% CI (0.47, 0.94)]. Elevated fibrinogen at 3 months was independently associated with a worse outcome on all clinical scales at 3 and 6 months [mRS: OR 1.63, 95% CI (1.12, 2.38) and OR 1.75, 95% CI (1.14, 2.70); BI: OR 0.62, 95% CI (0.42, 0.92) and OR 0.59, 95% CI (0.34, 0.88); MWS: OR 0.65, 95% CI (0.45, 0.93) and OR 0.61, 95% CI (0.40, 0.91)]. Elevated hs-CRP at 3 months was associated with a higher mRS (mRS ≥ 3) at 6 months [OR 1.89, 95% CI (1.02, 3.51)] but no independent associations of baseline hs-CRP with outcome were identified. TNF-alpha did not show any independent associations with investigated outcome parameters at follow-up.

**Table 3:** Associations of inflammatory biomarkers with outcome parameters in multivariate logistic regression models.

	**mRS**		**Barthel index**		**Mean walking speed**	
	**3 months** **after stroke**	**6 months** **after stroke**	**3 months** **after stroke**	**6 months** **after stroke**	**3 months** **after stroke**	**6 months** **after stroke**
**hs-CRP**						
Baseline [OR, (95% CI)]	1.16 (0.68, 1.98)	1.63 (0.88, 3.02)	0.90 (0.52, 1.56)	0.62 (0.34, 1.13)	0.60 (0.35, 1.03)	0.80;(0.45, 1.43)
3 months post-stroke [OR, (95% C)]	1.27 (0.73, 2.20)	1.89 (1.02, 3.51)^*^	0.64 (0.36, 1.14)	0.61 (0.34, 1.12)	0.75 (0.43, 1.29)	0.65 (0.36, 1.16)
**IL-6**						
Baseline [OR, (95% CI)]	2.04 (0.80, 5.22)	3.02 (1.01, 9.08)^*^	0.64 (0.25, 1.64)	0.36 (0.13. 1.02)	0.34 (0.13, 0.88)^*^	0.42 (0.15, 1.17)
3 months post-stroke [OR, (95% CI)]	1.54 (0.58, 4.07)	3.02 (0.88, 10.42)	0.47 (0.16, 1.38)	0.45 (0.15, 1.38)	0.70 (0.27, 1.80)	0.39 (0.12, 1.26)
**TNF-alpha**						
Baseline [OR, (95% CI)]	1.25 (0.13, 11.71)	2.23 (0.17, 29.65)	0.87 (0.09, 8.69)	1.51 (0.12, 18.71)	0.40 (0.04, 3.64)	0.17 (0.02. 2.02)
3 months post-stroke [OR, (95% CI)]	2.34 (0.31, 17.59)	2.46 (0.27, 22.69)	0.49 (0.06, 3.72)	0.22 (0.02, 2.21)	0.35 (0.50, 2.55)	0.24 (0.03, 2.16)
**Fibrinogen**						
Baseline [OR, (95% CI)]	1.36 (1.00, 1.86)	1.59 (1.08, 2.35)^*^	0.90 (0.66, 1.22)	0.66 (0.47, 0.94) ^*^	0.70 (0.51, 0.96)^*^	0.85 (0.61, 1.20)
3 months post-stroke [OR, (95% CI)]	1.63 (1.12, 2.38)^*^	1.75 (1.14, 2.70)^*^	0.62 (0.42, 0.92)^*^	0.59 (0.34, 0.88)^*^	0.65 (0.45, 0.93)^*^	0.61 (0.40, 0.91)^*^

* *p < 0.05*.

*Adjusted for: age, sex, NIHSS baseline, arterial hypertension*.

*hs-CRP, high-sensitive C-reactive protein; IL-6, Interleukin 6; TNF-alpha, Tumor Necrosis Factor alpha*.

## Discussion

In the presented study we analyzed consecutive serum levels of inflammatory markers of patients with subacute stroke. We observed upregulated inflammatory activity after stroke and a decline over a period of 6-months. Levels of investigated inflammatory markers were not modified in magnitude or dynamic by early aerobic physical fitness training. Elevated levels of IL-6 and fibrinogen in the early subacute phase showed associations with functional impairment up to 6-months.

### Inflammatory Markers

Hs-CRP is the marker that has received the most attention over the past decades when it comes to inflammatory biomarkers in stroke research. Many studies have underlined an elevation of hs-CRP after stroke and an association of higher levels with an unfavorable outcome (12). We found elevated hs-CRP after stroke followed by a significant decrease up to 6-months after the event. These findings are in line with previous studies describing an elevation of a panel of inflammatory markers in stroke patients (6, 7). However, in contrast to the literature, we could not show independent associations of hs-CRP levels with functional outcome parameters. One possible explanation is that the time point of blood draws differs from previous studies. Most studies focused on CRP blood levels in the acute phase after stroke or on measuring peak levels of CRP (12, 27, 28). Moreover, post-stroke infections are associated with higher levels of CRP, especially in the early phase after stroke (29). In our study, baseline visits varied from five to 45 (median 28 days) days post-stroke and acute phase reactions (including levels of CRP) most likely have already decreased.

There is growing evidence that elevated blood levels of IL-6 in acute stroke are associated with stroke lesion volume, stroke severity, post-stroke infection as well as worse short- and long-term outcome and death (1, 13–15, 30–32). Whiteley et al. underlined that adding blood values of IL-6 and NTproBNP to a validated score including age and NIHSS could improve outcome prediction (1, 14). Nevertheless, the impact was not substantial enough for this model to be useful in clinical practice. In contrast, the Linz Stroke Study claimed that the combination of several inflammatory markers can be a useful approach to predict post-stroke outcome in a clinical setting (15). Mouse models, however, showed a substantial role of IL-6 for angiogenesis after middle cerebral artery occlusion (17). No favorable effects of increasing IL-6 levels were stated in this analysis. Instead, we provide further evidence that upregulated IL-6 after stroke is correlated with worse functional outcome. We can specify the potential advantages of measuring IL-6 for prediction of impaired outcome, yet its clinical significance needs to be further validated.

There is good evidence that fibrinogen is involved in cardiovascular disease and negatively associated with clinical outcome (33–36). Evidence about the role of fibrinogen is still inconsistent and various studies were not able to show an independent impact on outcome (37). Moreover, adding fibrinogen to a model of age and NIHSS did not improve outcome prediction in stroke patients (38). Del Zoppo et al. showed that stroke patients with initial hyperfibrinogenemia had a worse outcome up to 90 days (39). Our study contributes further evidence of understanding the dynamics of fibrinogen after stroke and the advantages of measuring fibrinogen levels in clinical stroke management. In our cohort, fibrinogen levels remained elevated in the subacute phase of stroke and associations between higher inflammatory marker levels and impaired outcome were most profound for fibrinogen (Table 3). In addition, in contrast to other inflammatory markers, levels of fibrinogen over time were not associated with (common) pre-existing comorbidities, which makes it a more reliable marker to measure in the clinical setting.

Studies on TNF-alpha and stroke have yielded inconsistent results. Zaremba et al. indicated that elevated levels of TNF-alpha within 24 h of stroke-onset (in cerebrospinal fluid and serum) are correlated with lower BI scores up to 2 weeks (40). In contrast to these findings, Vila et al. were unable to show a significant correlation between raised serum values of TNF-alpha on admission and early neurological worsening up until 48 h after stroke (41). Our study expanded the knowledge to the subacute phase after stroke and could demonstrate that levels of TNF-alpha showed no dynamic change over the course of 6 months after stroke. We were not able to detect a specific expression pattern of TNF-alpha in the blood of patients after stroke. Moreover, we could not find any independent associations of serum TNF-alpha levels and functional outcome.

### Aerobic Fitness Training and Inflammatory Markers

Exercise potentially downregulates inflammatory activity in the long-term but only few interventional studies focus on its chronic effects on inflammatory markers. So far, study results either remain inconsistent or cannot show any favorable effects (42–44). In our cohort, fitness training did not have a substantial effect neither on levels of inflammatory biomarkers nor on clinical outcome. Furthermore, no significant effects of fitness training on primary outcome (MWS or BI) were observed in the main *PHYS-STROKE* study analyses (21). A possible reason for our lack of evidence might be that our aerobic fitness training sessions were not long and/or intense enough to initiate anti-inflammatory processes. Training-induced anti-inflammatory effects might be present only after longer, more intense training sessions. Given our study population with functional impairment post-stroke, longer training interventions could not have been performed.

### Strengths and Limitations

We investigated levels of inflammatory biomarkers within the *PHYS-STROKE* study, representing a multicenter, high quality trial. To our knowledge, most studies investigating post-stroke inflammatory markers focused on blood levels in the acute phase of stroke and only a few provide serial long-term measurements. In this study, however, we collected blood samples at four defined time-points until 6 months after stroke. We were able to depict the progression of inflammatory markers more precisely, even beyond the acute phase, covering the early and late subacute as well as early chronic phase. Additionally, we were able to examine a cohort with relatively severe impairments after stroke.

Certainly, this study has some limitations. Firstly, inclusion and baseline visit vary from a period of five to 45 days after the index stroke. This results in variances in the timing of the first intervention, as well as the post-intervention visit (v1). Blood levels of inflammatory markers might therefor vary between participants, with participants included at a later time-point post-stroke showing decreased inflammatory activity. Secondly, the effect that aerobic fitness training has on inflammatory markers might differ with different time points of training-initiation, resulting in us finding no significant effects in our cohort. Thirdly, we did not adapt for possible differences in activities outside the study intervention, as well as activities post-intervention. That could have influenced our study results. Moreover, we did not exclude patients with a diagnosis of infection or other conditions that lead to an upregulation of blood inflammatory markers.

### Impact and Future Research

Our study contributes to the literature on understanding the dynamics of inflammatory processes in stroke. In association with clinical outcome, the observed fluctuations can help to comprehend the role of inflammatory processes post-stroke. Including the measurement of inflammatory markers in routine stroke management can help identify patients that are at risk for long-term functional impairment. Models of outcome prediction including blood levels of inflammatory markers (single markers as well as a panel of markers) need to be validated in large, independent cohort studies. To understand the impact of fitness training on inflammation in stroke patients, researchers must first identify the ideal training program for counteracting inflammation. This research will support the important goal of identifying treatment options to blunt the neurotoxic effects of post-stroke inflammation in clinical practice. It remains unclear whether targeted anti-inflammatory treatment for stroke patients could prevent long-term stroke deterioration and this work is certainly deserving further research.

## Conclusion

Serum levels of hs-CRP, IL-6 and fibrinogen decrease up to 6 months following stroke. Aerobic fitness training did not modify levels of inflammatory markers compared to relaxation over time. Increased IL-6 and fibrinogen levels in early and late subacute stroke are associated with worse outcome up to 6-months after stroke.

## Data Availability Statement

The raw data will be made available upon reasonable request to the authors.

## Ethics Statement

The studies involving human participants were reviewed and approved by Institutional review board of Charité–Universitätsmedizin Berlin. The patients/participants provided their written informed consent to participate in this study.

## Author Contributions

AF, MEb, AN, and TR conceived or designed the study and supervised the study. BK drafted the manuscript and contributed in statistical analysis. TR, AF, MEb, and MEn critically revised the manuscript for important intellectual content and gave final approval of the version to be published.

## Conflict of Interest

MEn reports grants from Bayer and fees paid to the Charité from AstraZeneca, Bayer, Boehringer Ingelheim, BMS, Daiichi Sankyo, Amgen, GSK, Sanofi, Covidien, Novartis, Pfizer, all outside the submitted work. The remaining authors declare that the research was conducted in the absence of any commercial or financial relationships that could be construed as a potential conflict of interest.

## Publisher's Note

All claims expressed in this article are solely those of the authors and do not necessarily represent those of their affiliated organizations, or those of the publisher, the editors and the reviewers. Any product that may be evaluated in this article, or claim that may be made by its manufacturer, is not guaranteed or endorsed by the publisher.
