# Supplementary material for: Elevated Serum Inflammatory Markers in Subacute Stroke Are Associated With Clinical Outcome but Not Modified by Aerobic Fitness Training: Results of the Randomized Controlled PHYS-STROKE Trial
Source: Front Neurol. 2021 Aug 26;12:713018. doi: 10.3389/fneur.2021.713018 (PMC8426903; doi:10.3389/fneur.2021.713018)
Supplement: Supplementary file 1 [file Data_Sheet_1.PDF]

Supplementary Material

1 Supplementary Figures and Tables

1.1 Supplementary Tables

Supplementary Table 1. Inclusion and exclusion criteria to the PHYS-STROKE study

**Inclusion criteria**	
1.	Diagnosis of stroke (inclusion within 5-45 days after stroke onset); ischemic or hemorrhagic (cortical, subcortical, brainstem), as determined by initial MRI/CT scan of the brain)
2.	Age ≥ 18 years
3.	Able to sit unsupported (i.e. without holding onto supports such as the edge of the bed), with feet supported, for at least 30 seconds
4.	Barhtel-Index ≤ 65 at inclusion
5.	Considered able to perform aerobic exercise, as determined by responsible physician
6.	Provision of written informed content
**Exclusion criteria**	

1.	Patient considered unable to comply with study requirements
2.	Stroke due to intracranial haemorrhage primarily due to bleeding from ruptured aneurysm or arteriovenous malformation
3.	Progressive stroke
4.	Unable to perform the required exercises due to a) medical, b) musculo-skeletal, or c) neurological problems (for details see below, 4a-c)
4a.	Medical problems: unstable cardiovascular condition, or other serious cardiac conditions<br><br>(e. g., New York Heart Association criteria for Class IV heart disease, hospitalization for myocardial infarction or heart surgery within 120 days, severe cardiomyopathy or documented serious and unstable cardiac arrhythmias)
4b.	Musculoskeletal problems: restricted passive range of motion in the major lower limb joints (i.e. an extension deficit of > 20° for the affected hip or knee joints, or a dorsiflexion deficit of > 20° for the affected ankle)
4c.	Neurological problems: severity of stroke-related deficits
5.	Required help of at least 1 person to walk before stroke due to neurological (e. g., advanced Parkinson's disease, Amyotrophic Lateral Sclerosis, Multiple Sclerosis) or non-neurological<br><br>co-morbidities (e. g. heart failure, orthopaedic problems)
6.	Life expectancy of less than 1 year as determined by responsible physician
7.	Drug or alcohol addiction within the last six months
8.	Significant current psychiatric illness defined as medication-refractory of bipolar affective disorder, psychosis, schizophrenia or suicidality

9.	Current participation in another interventional trial

MRI = magnetic resonance imaging

CT = computer tomography

Supplementary Table 2. Mean values of inflammatory biomarkers at follow-up visits

	Baseline	Baseline + 4 weeks	3 months after stroke	6 months after stroke
**hs-CRP (mg/l, mean ± SD) ***	12.04 ±18.72	7.15 ±11.23	7.11 ±14.86	4.94 ±9.94
**IL-6 (pg/ml, mean ± SD)** [+]	6.34 ±11.72	6.13 ±21.77	6.92 ±31.31	4.07 ±8.11
**TNF-alpha (pg/ml, mean ± SD)** [§]	9.34 ±3.85	9.33 ±4.35	9.11 ±4.06	8.56 ±3.53
**Fibrinogen (g/l, mean ± SD)** [X]	4.09 ±1.15	3.78 ±0.87	3.8 ±0.97	3.55 ±0.8

* normal range <3.0 mg/l

[+] normal range <3.8 pg/ml

[§] normal range <8.1 pg/ml

[X] normal range 2-4 g/l

hs-CRP: high-sensitive C-reactive protein; IL-6: Interleukin 6; TNF-alpha: Tumor Necrosis Factor alpha

Supplementary Table 3. Median outcome parameters at clinical follow-up visits

	Baseline	Baseline + 4 weeks	3 months after stroke	6 months after stroke
**mRS (median [IQR]))**	4 (4-4)	4 (3-4)	3 (2-4)	3 (2-4)
**BI (median [IQR])**	50 (35-60)	75 (60-90)	80 (65-95)	90 (70-100)
**MWS (m/S, median [IQR])**	0.30 (0.13-0.66)	0.51 (0.25-0.77)	0.57 (0.18-1.00)	0.83 (0.35-1.25)

mRS: modified Rankin Scale, BI: Barthel Index, MWS: Mean walking speed

Supplementary Table 4. Associations of inflammatory biomarkers with outcome parameters in univariate logistic regression models

	**mRS**		**Barthel Index**		**Mean Walking Speed**	
	3 months post-stroke	6 months after stroke	3 months after stroke	6 months after stroke	3 months after stroke	6 months after stroke
**hs-CRP**						
Baseline (OR [95% CI])	1.32, (0.80, 2.20)	1.87, (1.05, 3.31)*	0.75, (0.45, 1.24)	0.53, (0.30, 0.93)*	0.55, (0.32, 0.94)*	0.70, (0.40, 1.21)
3 months post-stroke (OR [95% CI])	1.39, (0.83, 2.31)	1.89 (1.08, 3.32)*	0.57, (0.34, 0.97)*	0.61 (0.35, 1.06)	0.69, (0.41, 1,14)	0.56 (0.31, 0.98)*
**IL-6**						
Baseline (OR [95% CI])	2.31, (0.98, 5.45)	4.21, (1.54, 11.51)*	0.45, (0.19, 1.06)	0.24, (0.09, 0.63)*	0.33, (0.13, 0.81)*	0.28, (0.11, 0.74)*
3 months post-stroke (OR [95% CI])	1.78, (0.76, 4.20)	3.32 (1.15, 9.62)*	0.35, (0.14, 0.89)	0.35 (0.13, 0.92)*	0.64, (0.27, 1.50)	0.29 (0.10, 0.84)*
**TNF-alpha**						
Baseline (OR [95% CI])	1.20, (0.17, 8.68)	2.76., (0.31, 24.36)	0.62, (0.09, 4.55)	0.65, (0.08, 5.33)	0.49, (0.07, 3.71)	0.10, (0.01, 0.91)*
3 months post-stroke (OR [95% CI])	2.57, (0.45, 14.78)	3.68 (0.50, 27.04)	0.29, (0.05, 1.71)	0.23 (0.03, 1.70)	0.37, (0.06, 2.22)	0.15 (0.02, 1.18)
**Fibrinogen**						
Baseline (OR [95% CI])	1.40, (1.04, 1.88)*	1.66, (1.16, 2.35)*	0.85, (0.64, 1.12)	0.62, (0.44, 0.86)*	0.68, (0.50, 0.92)*	0.82, (0.60, 1.13)
3 months post-stroke (OR [95% CI])	1.60, (1.13, 2.71)**	2.00 (1.29, 3.10)**	0.62, (0.44, 0.89)**	0.55 (0.37, 0.82)**	0.64, (0.45, 0.91)*	0.51 (0.33, 0.79)**

***p < 0.05**

****p≤0.01**

hs-CRP: high-sensitive C-reactive protein; IL-6: Interleukin 6; TNF-alpha: Tumor Necrosis Factor alpha; mRS: modified Rankin Scale

Supplementary Table 5. Associations of inflammatory biomarkers with outcome parameters in multivariate logistic regression models; adjusted for age, sex and NIHSS at baseline

	**mRS**		**Barthel Index**		**Mean Walking speed**	
	3 months post-stroke	6 months after stroke	3 months after stroke	6 months after stroke	3 months after stroke	6 months after stroke
**hs-CRP**						
Baseline (OR, [95% CI])	1.16 (0.68, 1.98)	1.63 (0.88, 3.02)	0.90 (0.52, 1.56)	0.62 (0.34, 1.13)	0.60 (0.35, 1.03)	0.82 (0.46, 1.46)
3 months post-stroke (OR, [95% CI])	1.23 (0.73, 2.20)	1.89 (1.02, 3.51)*	0.64 (0.36, 1.14)	0.61 (0.34, 1.12)	0.73 (0.43, 1.25)	0.65 (0.36, 1.16)
**IL-6**						
Baseline (OR, [95% CI])	1.04 (0.80, 5.22)	3.02 (1.01, 9.08)*	0.64 (0.25, 1.64)	0.36 (0.13, 1.02)	0.34 (0.13, 0.88) *	0.42 (0.15, 1.17)
3 months post-stroke (OR, [95% CI])	1.54 (0.58, 4.01)	2.99 (0.87, 10.19)	0.47 (0.16, 1.38)	0.45 (0.15, 1.38)	0.69 (0.27, 1.78)	0.39 (0.12, 1.26)
**TNF-alpha**						
Baseline (OR, [95% CI])	1.35 (0.13, 11.71)	2.47 (0.19, 32.14)	0.88 (0.09, 8.73)	1.18 (0.10, 13.94)	0.38 (0.04, 3.43)	0.17 (0.02, 2.02)
3 months post-stroke (OR, [95% CI])	2.34 (0.31, 17.59)	2.61 (0.29, 23.82)	0.49 (0.06, 3.72)	0.22 (0.02, 2.21)	0.35 (0.05, 2.55)	0.24 (0.03, 2.16)
**Fibrinogen**						
Baseline (OR, [95% CI])	1.34 (1.00, 1.85)	1.59 (1.08, 2.35)*	0.90 (0.66, 1.22)	0.66 (0.47, 0.94)*	0.70 (0.51, 0.96) *	0.87 (0.62, 1.21)
3 months post-stroke (OR, [95% CI])	1.63 (1.12, 2.38) *	1.75 (1.14, 2.70)*	0.62 (0.42, 0.92)*	0.59 (0.40, 0.88)*	0.64 (0.44, 0.92) *	0.61 (0.40, 0.91)*

***p < 0.05**
Adjusted for: age, sex, NIHSS baseline

hs-CRP: high-sensitive C-reactive protein; IL-6: Interleukin 6; TNF-alpha: Tumor Necrosis Factor alpha

1.2 Supplementary Figures

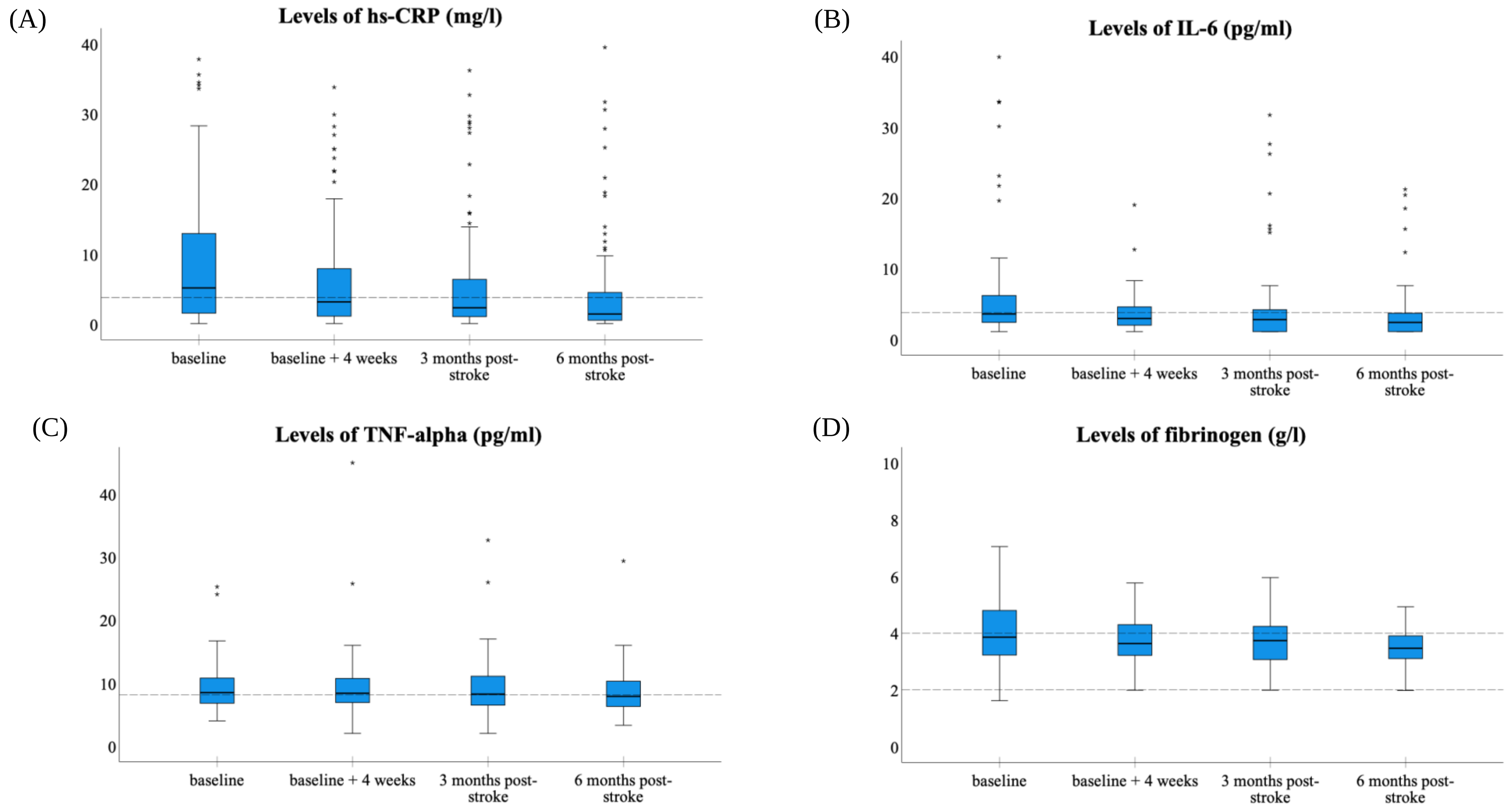

Supplementary Figure 1. Median levels of inflammatory biomarkers over time in the *PHYS-STROKE* study cohort; hs-CRP (A), IL-6 (B), TNF-alpha (C), fibrinogen (D); horizontal dashed lines depict cut-off for normal ranges
